# Whole genome sequencing of Ethiopian *Brucella abortus* isolates expands the known diversity of an early branching sub-Saharan African lineage

**DOI:** 10.3389/fmicb.2023.1128966

**Published:** 2023-05-04

**Authors:** Bedaso Mammo Edao, Gobena Ameni, Stefan Berg, Muluken Tekle, Adrian M. Whatmore, James L. N. Wood, Andries J. van Tonder, Roland T. Ashford

**Affiliations:** ^1^Department of Veterinary Medicine, University of Cambridge, Cambridge, United Kingdom; ^2^College of Veterinary Medicine, Addis Ababa University, Bishoftu, Ethiopia; ^3^Aklilu Lemma Institute of Pathobiology, Addis Ababa University, Addis Ababa, Ethiopia; ^4^Department of Veterinary Medicine, College of Food and Agriculture, United Arab Emirates University, Al Ain, United Arab Emirates; ^5^Department of Bacteriology, Animal and Plant Health Agency, Weybridge, United Kingdom

**Keywords:** *Brucella abortus*, sub-Saharan Africa, lineage, whole genome sequencing, molecular typing

## Abstract

Brucellosis remains one of the most significant zoonotic diseases globally, responsible for both considerable human morbidity and economic losses due to its impacts on livestock productivity. Despite this, there remain significant evidence gaps in many low- and middle-income countries, including those of sub-Saharan Africa. Here we report the first molecular characterisation of *Brucella* sp. from Ethiopia. Fifteen *Brucella* sp. isolates from an outbreak in cattle from a herd in central Ethiopia were identified as *Brucella abortus*, using bacterial culture and molecular methods. Sequencing of the Ethiopian *B. abortus* isolates allowed their phylogenetic comparison with 411 *B. abortus* strains of diverse geographical origins, using whole genome single nucleotide polymorphisms (wgSNP). The Ethiopian isolates belonged to an early-branching lineage (Lineage A) previously only represented by data from two strains, both of sub-Saharan African origin (Kenya and Mozambique). A second *B. abortus* lineage (Lineage B), also comprised solely of strains originating from sub-Saharan Africa, was identified. The majority of strains belonged to one of two lineages of strains originating from a much broader geographical range. Further analyses based on multi-locus sequence typing (MLST) and multi-locus variable-number tandem repeat analysis (MLVA) expanded the number of *B. abortus* strains available for comparison with the Ethiopian isolates and were consistent with the findings from wgSNP analysis. MLST profiles of the Ethiopian isolates expanded the sequence type (ST) diversity of the early branching lineage of *B. abortus*, equivalent to wgSNP Lineage A. A more diverse cluster of STs, equivalent to wgSNP Lineage B, was comprised solely of strains originating from sub-Saharan Africa. Similarly, analysis of *B. abortus* MLVA profiles (*n* = 1891) confirmed that the Ethiopian isolates formed a unique cluster, similar to only two existing strains, and distinct from the majority of other strains of sub-Saharan African origin. These findings expand the known diversity of an under-represented lineage of *B. abortus* and suggest a potential evolutionary origin for the species in East Africa. In addition to providing information concerning *Brucella* species extant within Ethiopia this work serves as the basis for further studies on the global population structure and evolutionary history of a major zoonotic pathogen.

## 1. Introduction

Brucellosis is a zoonotic infection caused by bacteria of the genus *Brucella*, which affects domestic livestock and a wide range of wild mammals (Ducrotoy et al., 2017). The disease is amongst the most common zoonotic infections globally, with an estimated 500,000 human cases annually (Pappas et al., 2006), though this figure is likely to be a significant under-estimate of the burden of disease (Moreno et al., 2022). Brucellosis remains endemic in much of Africa, South America, the Middle East and the Mediterranean region of Europe (Pappas et al., 2006; McDermott et al., 2013). However, there is a notable lack of evidence concerning endemic brucellosis in many low-income countries of Africa and Asia (McDermott et al., 2013).

Infection in humans occurs primarily as a result of direct contact with infected animals (in particular their products of conception), or ingestion of unpasteurised dairy products from infected animals (Corbel, 2006). Human brucellosis is characterized by febrile illness which can lead to debilitating chronic conditions if left untreated (Dean et al., 2012). In addition, brucellosis has indirect health consequences, especially for livestock-keeping populations in resource-limited settings, which depend on livestock for food security and income (McDermott et al., 2013; Lokamar et al., 2020).

The majority of human brucellosis infections are caused by two *Brucella* species, *Brucella melitensis* and *Brucella abortus*, which exhibit marked livestock host preferences (Corbel, 2006). Brucellosis in small ruminants (sheep and goats) is primarily caused by *B. melitensis*, whilst in cattle the infection is most commonly caused by *B. abortus*. However, in areas where cattle are kept in close association with sheep or goats, as is common in many mixed-livestock keeping populations, infection in cattle may also be caused by *B. melitensis*, and in small ruminants by *B. abortus* (e.g., Akoko et al., 2021).

Developments in molecular typing have contributed to current understanding of the global population structure of *Brucella* spp. organisms and of local disease epidemiology (Whatmore and Foster, 2021; Ashford and Whatmore, 2022). Two methods, in particular, have been applied to explore genetic relationships between *Brucella* species, and relate these to the geographic origin of strains; multi-locus sequence typing (MLST) and multi-locus variable number tandem repeat analysis (MLVA). Whatmore et al. (2016) used a 21-locus MLST scheme (extending the existing 9-locus scheme; Whatmore et al., 2007) to identify three major clades within *B. abortus* (referred to as A, B and C). In this dataset clades A and B were comprised entirely of isolates of sub-Saharan African origin (Chad, Kenya, Mozambique, Nigeria, Sudan, Uganda and Zimbabwe), whilst clade C (further sub-divided into C1 and C2) originated from a broad geographical range and contained the majority of isolates for which data were available. Clade A, the most basal in the *B. abortus* MLST phylogeny, was represented by just two historical strains, isolated from Kenya and Mozambique in 1963 and 1988, respectively. It was suggested that these findings indicate the existence of substantial diversity yet to be characterised in *B. abortus* isolates from Africa, which are significantly under-represented in molecular typing databases.

Multi-locus variable-number tandem repeat analysis loci are not typically employed for inferring ancestral phylogenetic relationships, due to their higher mutation rates and greater risk of homoplasy (e.g., Keim et al., 2004). Nonetheless, global analyses of *B. abortus* population structure based on MLVA have supported findings from MLST studies. For example, a large analysis by Vergnaud et al. (2018) described three *B. abortus* clades which correspond broadly with the clades B, C1 and C2 described above. Clade B was comprised solely of isolates originating in Africa, with the majority of these isolated in West Africa (Cameroon, Guinea, Guinea-Bissau, Mauritania, Niger, Senegal, Togo) and a smaller number from the east of the continent (Rwanda and Sudan). Clade A was absent from this analysis, which did not include data from the two basally located strains reported by Whatmore et al. (2016).

Whole genome sequencing (WGS) methods are increasingly being applied to describe relationships between *Brucella* strains, either replacing established molecular typing techniques or augmenting studies based on existing approaches (e.g., MLST) and extracting these data *in silico* (Whatmore and Foster, 2021; Ashford and Whatmore, 2022). Ledwaba et al. (2021) reported *B. abortus* isolates from various regions of South Africa, using whole-genome single nucleotide polymorphisms (wgSNPs) to describe relationships with publicly available genomes. Consistent with previous results, three major lineages (referred to as A, B and C) were identified, with the South African isolates clustering in lineage C, with isolates from a broad geographic range, including Africa (Mozambique and Zimbabwe), Europe, Asia and America. Other recent studies reporting whole genome sequencing of *B. abortus* from North Africa have focused primarily on local disease epidemiology rather than broader population structure (Holzer et al., 2021; Khan et al., 2021).

In Ethiopia, studies from various regions of the country have reported individual-level brucellosis prevalence ranging from 0.06% in commercial intensive dairy production (Edao et al., 2018) to 9.7% in an extensive production system at the livestock-wildlife interface (Chaka et al., 2018). A recent systematic review and meta-analysis reported an estimated seroprevalence of 2.6% (95% CI: 2.2–3.0) in cattle, which increased to 16.3% (95% CI: 12.9–20.5) when herd-level prevalence was estimated (Sibhat et al., 2022). However, only two studies have reported the isolation of *Brucella* sp. strains from Ethiopia. Tekle et al. (2019) reported the isolation of *B. melitensis* from goats in the Afar region of eastern Ethiopia, and Geresu et al. (2016) reported the isolation of *B. abortus* from dairy cattle in central Ethiopia. To date there have been no studies reporting the molecular typing of *Brucella* sp. from Ethiopia.

There remains a dearth of *B. abortus* strains originating from sub-Saharan Africa (SSA), and WGS data from this region are consequently significantly under-represented in public sequence databases. To date there have been no published global analyses of *B. abortus* integrating data from both WGS and existing molecular typing methods (MLST and MLVA). In this study, we report the first isolation and molecular characterisation of Ethiopian *B. abortus* strains. We apply existing molecular typing approaches and whole genome sequence data analysis in order to characterise the strains and their phylogenetic placement relative to the global diversity of this important zoonotic pathogen.

## 2. Materials and methods

### 2.1. Study location, animals and sample collection

**Figure 1:**
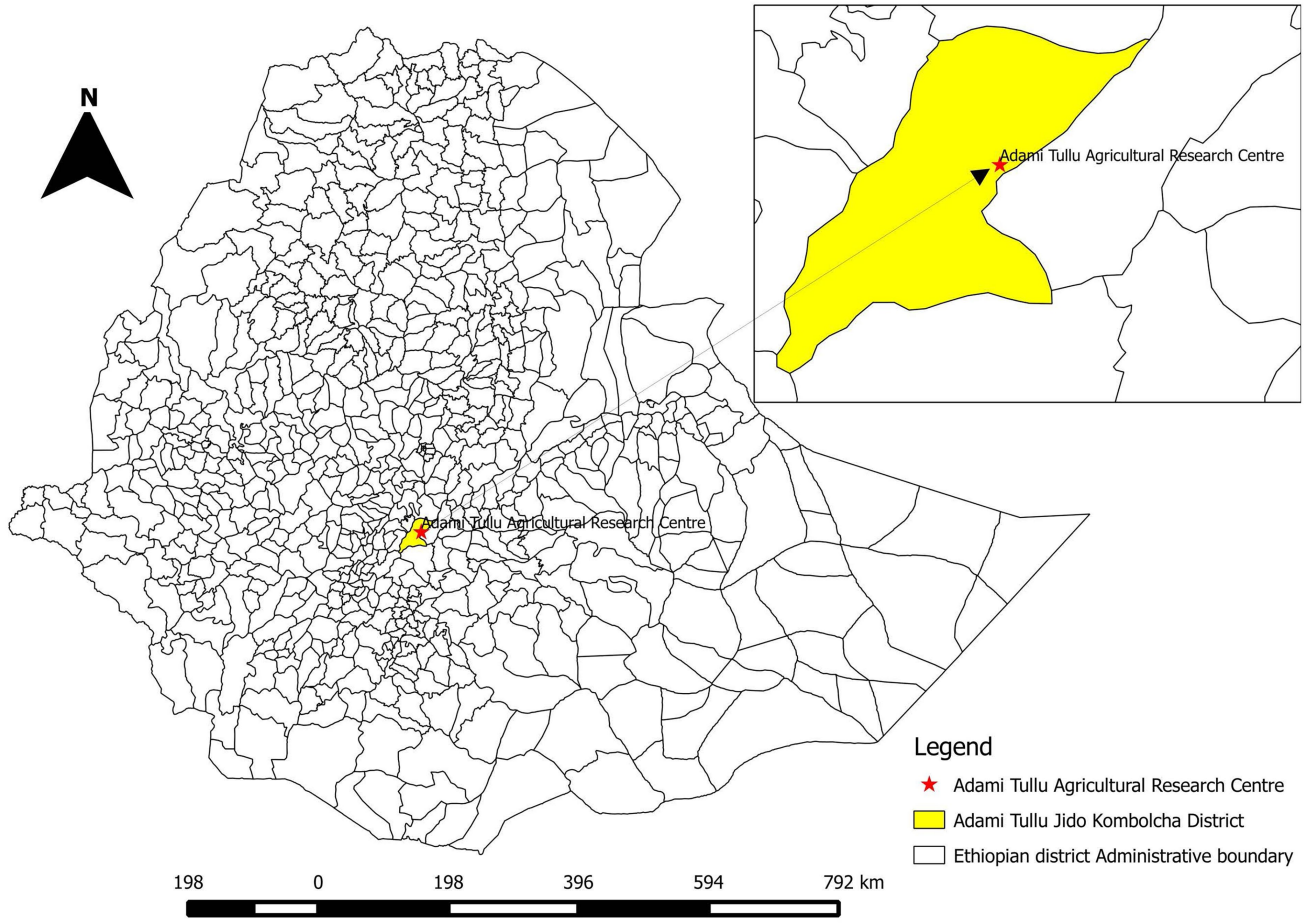
Map of Ethiopia, showing Adami Tullu Jido Kombolcha District and the location of Adami Tullu Agricultural Research Centre (ATARC).

Adami Tullu Agricultural Research Centre (ATARC) is located in the mid Rift Valley, central Ethiopia, 167 km south of Addis Ababa in Oromia National Regional State (Figure 1). It lies at latitude 7°9’N and longitude 38°7′E at an elevation of 1,650 m above sea level. The study population included a total of 547 Jersey or Holstein Friesian (*Bos taurus*), Arsi zebu (*Bos indicus*) and crossbred (*Bos indicus* × *Bos taurus*) dairy cattle, which were reared semi-intensively at ATARC. A high number of abortions within the ATARC herd were reported to the Assela Veterinary Regional Laboratory in March 2018, and consequently an investigation of the outbreak was conducted (Edao, 2020).

Seropositive animals were identified using serial testing by Rose Bengal test (RBT) and competitive ELISA (cELISA; Table 1), performed according to standard protocols (WOAH, 2018). Of 547 animals in the ATARC herd 125 (22.85%) were identified as seropositive by both RBT and cELISA (Table 1). A sub-sample of seropositive animals with a history of abortion were opportunistically selected during culling for *post mortem* sample collection. From these 30 animals, 66 samples were collected for bacteriological culture, comprising 28 vaginal swabs, 24 uterine tissue samples, 13 mammary gland lymph node samples and a single placental sample. A vaginal swab was available for 28 animals; 23 also had a uterine tissue sample, ten additionally had a mammary gland lymph node sample and in two cases a mammary gland lymph node sample was available but uterine tissue was absent. The two animals missing swab samples had, respectively, one (placenta) and two (mammary gland lymph node and uterine tissue) other samples.

**Table 1:** Summary data for 125 seropositive cattle identified by Rose Bengal test (RBT) and competitive ELISA (cELISA) during the suspected brucellosis outbreak investigation at Adami Tullu Agricultural Research Centre (ATARC), central Ethiopia.

	Category	Number of animals
Breed	Jersey	1
	Jersey × Arsi	1
	Holstein Friesian × Arsi	15
	Arsi	108
Age	≤ 3 years	25
	> 3 years	100
Number of pregnancies	≤ 2	108
	> 2	17
Reproductive status	Pregnant	50
	Not pregnant	75
Breeding	Natural	6
	Artificial insemination	119
Number of abortions recorded	One	63
	Two	62

Tissue samples (uterus, mammary gland lymph nodes and placenta) were collected into 20 ml sterile centrifuge tubes with sterile saline. Vaginal swabs were collected using Amies sterile media swabs (Deltalab, Spain). Thereafter, all samples were transported with ice packs to the Aklilu Lemma Institute of Pathobiology, Addis Ababa University (ALIPB-AAU) where they were stored at −20°C until shipped to the WOAH Brucellosis Reference Laboratory and FAO Reference Centre for Brucellosis at the Animal and Plant Health Agency (APHA) in the United Kingdom.

### 2.2. *Brucella* isolation and culture

Bacteriological confirmation was performed using standard protocols (Alton et al., 1975), at the Animal and Plant Health Agency (APHA), United Kingdom, between August 2018 and October 2018. A total of 66 samples consisting of 28 vaginal swabs and 38 tissue samples were cultured. The tissue samples were manually trimmed then macerated using a mechanical homogeniser (Stomacher Bagmixer 100 MiniMix, Seward Ltd, United Kingdom). The tissue suspensions and swab samples were inoculated directly onto both Farrell’s and serum dextrose agar (SDA) media plates. All plates were incubated with 10% CO_2_ at 37°C for three to five days. One colony was selected for sub-culture on Farrell’s media for further molecular identification and typing. Pure colonies were harvested in 500 μL nuclease-free water and inactivated at 100°C for 10 min, to produce thermo-lysates.

### 2.3. Molecular identification of isolates

The identity of isolates was initially confirmed using *Brucella* spp. specific quantitative PCR (qPCR) assays based on insertion sequence IS*711* and *bcsp*31 targets (Probert et al., 2004; Matero et al., 2011, respectively). The qPCR assays were considered positive when amplification was observed at cycle threshold (Ct) values of ≤ 35 for IS*711* and ≤ 40 for *bcsp*31. Identification to species level was performed using the Bruceladder multiplex PCR (Mayer-Scholl et al., 2010; López-Goñi et al., 2011) and qPCR-based single nucleotide polymorphism (SNP) typing (Gopaul et al., 2008). For all assays, thermo-lysates, prepared as described above, were used.

### 2.4. Whole genome sequencing

*Brucella* sp. cultures identified as described above were subsequently submitted for whole genome sequencing. Thermo-lysates were pelleted by centrifugation (10,000 *g* for 10 min), prior to DNA extraction using the Qiagen DNeasy Blood and Tissue Kit (Qiagen, United Kingdom) following the manufacturer’s protocol for Gram-negative bacteria. DNA concentrations were quantified using the Qubit 2.0 fluorometer and Qubit dsDNA HS (High Sensitivity) Assay Kit (Thermo Fisher Scientific, United Kingdom). Genomic libraries were constructed using the NEBNext Ultra II DNA Library Prep Kit for Illumina (New England Biolabs Inc., United Kingdom) according to the manufacturer’s instructions. The library size selection was 550 base pairs (bp) and a 250 bp paired-end (PE) sequencing strategy was employed using the MiSeq platform and MiSeq Reagent Kit V2 (Illumina Inc., San Diego, CA, United States), following the manufacturer’s recommended protocol. Basic quality control metrics for the raw sequence data were generated using FastQC^1^ and the reads were trimmed using fastp (Chen et al., 2018) to remove low quality reads and adapter sequences.

### 2.5. Whole genome sequencing data collection and quality assessment

Genome data downloaded from NCBI^2^ and ENA^3^ were manually checked and duplicate entries removed. This included reference and vaccine strains sequenced by multiple institutions, as well as strains present in both assembly and short-read data formats. Sequence reads with similarity to *Brucella* species were identified using Kraken 2 (Wood et al., 2019) and Bracken (Lu et al., 2017) and samples with < 70% reads assigned to *B. abortus* were excluded from further analyses.

### 2.6. Whole genome SNP (wgSNP) analysis

The *B. abortus* biovar 1 reference strain 9-941 (RefSeq accession number: GCF_000008145.1) was used as the reference genome for mapping of the Ethiopian sequences and NCBI/ENA data. *B. abortus* 9-941 is a well characterised reference strain which was the first published genome sequence for the species (Halling et al., 2005). The genome is a complete assembly (NCBI accession numbers NC_006932 and NC_006933 for chromosome I and II respectively) which has previously been used as a reference in several published whole genome sequencing studies (e.g., Suarez-Esquivel et al., 2020). The bactmap pipeline^4^ was used for mapping and variant calling. Briefly, sequence data were mapped to the reference with BWA mem (Li and Durbin, 2009), variants were called and filtered with BCFtools (Li, 2011) and consensus fasta sequences were generated for each sample using a python script. The consensus fasta sequences and the reference sequence were then used to create a multiple sequence alignment from which the variant sites were extracted using SNP-sites (Page et al., 2016). Samples that mapped to < 75% of the reference genome were omitted from further analyses. The resulting alignment of variable SNP sites was then used to construct a maximum likelihood phylogeny with IQ-TREE (Nguyen et al., 2014) using the model finder (MFP) and 1,000 fast bootstraps. The phylogeny was rooted using *B. melitensis* type strain 16M^T^ (GCF_000007125.1) and visualised and annotated using the R library ggtree (Yu et al., 2017; R Core Team, 2022). Pairwise SNP distances for all genomes were calculated using pairsnp.^5^

### 2.7. *In silico* MLST and MLVA

Ethiopian *B. abortus* isolates and *B. abortus* fastq files downloaded from the ENA were *de novo* assembled using SPAdes v3.13.1 (Bankevich et al., 2012). The quality of the final assemblies was assessed using Quast (Gurevich et al., 2013).

Assembled genomes of the Ethiopian *Brucella* isolates underwent *in silico* multi-locus sequence typing (mlst)^6^ to retrieve both nine and 21 locus allelic profiles (Whatmore et al., 2007, 2016). *In silico* MLST was also performed on *B. abortus* genome assemblies retrieved from NCBI and short-read data from ENA (following *de novo* assembly). Additionally, *B. abortus* allelic profiles from the *Brucella* PubMLST database^7^ were downloaded for inclusion in the analysis. This database includes MLST profiles generated using both Sanger sequencing and *in silico* MLST typing from WGS data.

*In silico* 16-locus MLVA (MLVA16: Le Fleche et al., 2006) typing was undertaken using a purpose-written script (MLVA_finder)^8^ applied to genome assemblies, as described by Vergnaud et al. (2018). Genotypes obtained by *in silico* MLVA16 analysis were compared with entries for *B. abortus* accessed via the publicly accessible *Brucella* MLVA database^9^ (Brucella v4_6_3). This database includes MLVA profiles generated using both traditional fragment sizing approaches and *in silico* MLVA typing from WGS data (Vergnaud et al., 2018). The Hunter-Gaston diversity index (HGDI) was used to describe the discriminatory capacity of MLVA loci for Ethiopian isolates (Hunter and Gaston, 1988).

Molecular typing data downloaded from PubMLST and MLVA databases, respectively, were manually checked and duplicate entries removed. This included reference and vaccine strains sequenced by multiple institutions, as well as field isolates for which both conventional (Sanger sequencing) data and *in silico* typing data were present. Allelic profiles and associated metadata for both MLST and MLVA were visualised with minimum spanning trees, using GrapeTree (Zhou et al., 2018).

## 3. Results

### 3.1. *Brucella* culture and molecular identification

Of 66 bovine tissue and swab samples processed for isolation of *Brucella* spp., 15 samples (22.7%) were culture positive (nine mammary gland lymph nodes, three uterine tissues and three vaginal swabs). Table 2 summarises the samples from which putative *Brucella* sp. cultures were recovered. Thermo-lysates from cultures generated Ct values between 13.05 and 14.36 for IS*711* and 15.44 and 17.04 for *bcsp*31, confirming that all isolates belong to the genus *Brucella* (see Supplementary Table S1). Bruceladder multiplex PCR identified all 15 isolates as *B. abortus* based on the pattern of amplified products observed following electrophoresis (products corresponding to 1,682, 794, 587, 450 and 152 bp in length; see Supplementary Table S1). Similarly, qPCR-based SNP assays identified all isolates as *B. abortus* (see Supplementary Table S1).

**Table 2:** Details of *Brucella abortus* isolates from Ethiopian cattle included in the current study, with NCBI SRA accession numbers.

Isolate ID	Sequence ID	Animal ID	Sample type	Accession #
F5/18-T8	1_S1_L001	1968	Mammary gland LN	ERS5240204
F5/18-T6	2_S2_L001	1678	Mammary gland LN	ERS5240211
F5/18-T5	3_S3_L001	2016	Mammary gland LN	ERS5240212
F5/18-T10	4_S4_L001	1966	Mammary gland LN	ERS5240213
F5/18-T7	5_S5_L001	2034	Mammary gland LN	ERS5240214
F5/18-T4	6_S6_L001	2079	Mammary gland LN	ERS5240215
F5/18-T3	7_S7_L001	1681	Mammary gland LN	ERS5240216
F5/18-S35	8_S8_L001	2133	Vaginal swab	ERS5240217
F5/18-S14	9_S9_L001	1540	Vaginal swab	ERS5240218
F5/18-T18	10_S10_L001	2115	Uterine tissue	ERS5240205
F5/18-T15	11_S11_L001	2032	Mammary gland LN	ERS5240206
F5/18-T21	12_S12_L001	2108	Uterine tissue	ERS5240207
F5/18-T16	13_S13_L001	1672	Uterine tissue	ERS5240208
F5/18-S36^1^	14_S14_L001	2144	Vaginal swab	ERS5240209
F5/18-T12^1^	15_S15_L001	2144	Mammary gland LN	ERS5240210

LN = lymph node. ^1^Isolates F5/18-S36 and F5/18-T12 from vaginal swab and mammary gland lymph node from the same animal.

### 3.2. Whole genome sequencing, *de novo* assembly and quality assessment

An average of 2,787,993 reads per sample were generated by Illumina sequencing of the Ethiopian *B. abortus* isolates. Summary statistics for Illumina sequencing and mapping for each isolate are provided in Supplementary Table S2. *De novo* assembly of the Ethiopian sequence data resulted in an average of 38 contigs per genome, an average total genome length of 3,273,080 bp, an average GC content of 57.23% and an average N50 of 389,770. Summary statistics for each *de novo* genome assembly are provided in Supplementary Table S2.

Five NCBI genome assemblies were removed from further analysis following identity assessment by Kraken/Bracken, due to the low proportion of reads identified as originating from *B. abortus* (< 70%). For two of these assemblies the majority of reads were identified as *Brucella suis* (strains BCB013 [GCA_000292205] and B104M [GCA_000292045]), whilst for three the majority of reads were identified as *B. melitensis* (strains S-586 [GCA_016091965], 2308 [GCA_018604785] and BCB027 [GCA_000292145]). Following mapping, 84 SRA datasets were removed due to the relatively low proportion of the reference genome mapped by the reads (< 75%). These SRA datasets included 72 from the United States, seven from Costa Rica and five from Egypt.

### 3.3. Whole genome SNP (wgSNP) analysis

Phylogenetic analysis was undertaken using a total of 426 *B. abortus* whole genome sequences. In addition to data from 15 Ethiopian isolates this included 153 assembled genomes and 258 short read datasets, downloaded from NCBI and ENA, respectively. Accession numbers for all downloaded sequences can be found in Supplementary Table S3. Mapping of Ethiopian *B. abortus* sequence data against reference strain 9–941 demonstrated that all Ethiopian *B. abortus* isolates differed by at least 3,749 SNPs from the chosen reference strain. Within the Ethiopian *B. abortus* isolates there was relatively little diversity evident in wgSNP analysis, with no more than five SNPs identified between any two strains within the panel (0–5). A matrix of pairwise SNP distances between all strains incorporated in the wgSNP analysis is provided in Supplementary Table S4.

**Figure 2:**
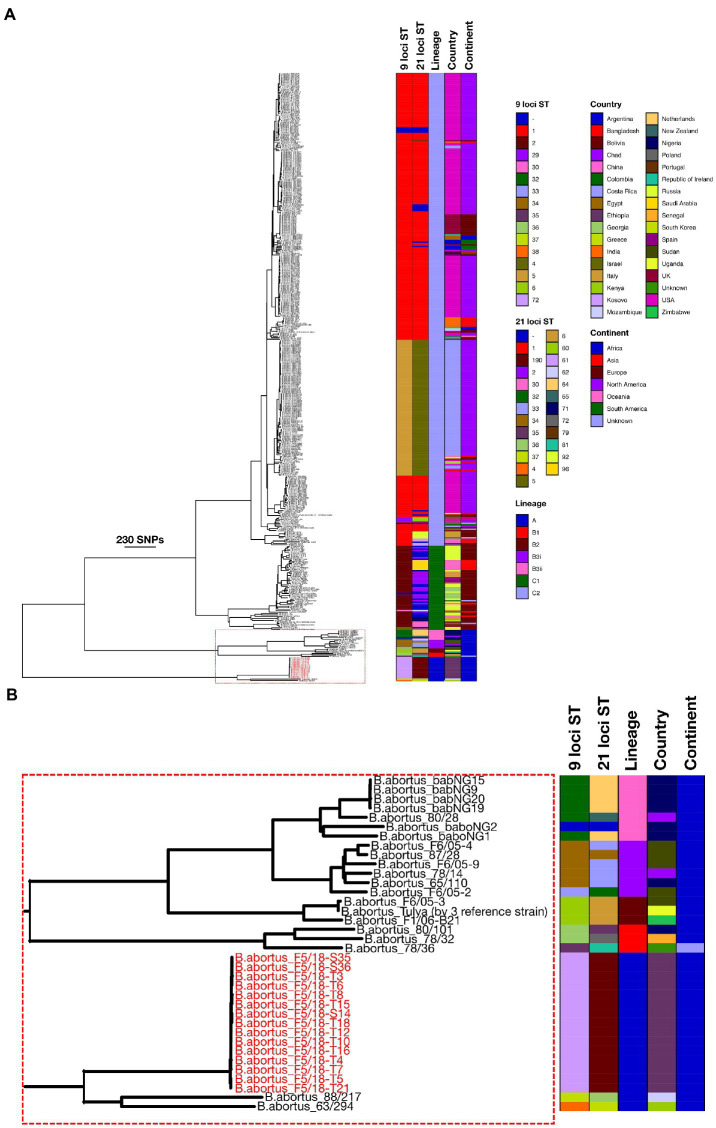
Maximum likelihood phylogeny of *Brucella abortus* strains (*n* = 426), including 15 Ethiopian isolates from the present study (tip labels highlighted in red), based on wgSNPs. Colour-coded keys give 9- and 21-locus sequence type (ST), continent and country of origin, and lineage. The scale bar shows number of SNPs. *B. melitensis* (16M^T^) was used to root the phylogenetic tree (not shown). Figure 2B shows the section of the phylogeny containing the Ethiopian isolates and other strains in Lineages A and B.

Phylogenetic analysis based on wgSNPs demonstrated that *B. abortus* from Ethiopia clustered with *B. abortus* strains from Mozambique (strain 88/217) and Kenya (strain 63/294; Figure 2; Lineage A). These two strains, along with the 15 Ethiopian isolates, formed a distinct lineage, basal to all other *B. abortus* strains included in the current analysis. Strains 88/217 and 63/294 differed from the Ethiopian isolates by 828 to 834 and 802 to 808 SNPs respectively, and by 698 SNPs from each other.

Outside of this basally branching lineage, a second lineage contained a larger number of isolates, also of exclusively sub-Saharan African origin (Figure 2; Lineage B), though a single strain in this lineage did not have a geographic origin recorded in the available metadata. Within Lineage B three isolates formed a basal cluster (Figure 2; Lineage B1). These three strains originated from Nigeria (strain 80/101), Senegal (strain 78/32) and an unknown location (strain 78/36). Outside of Lineage B1 a second lineage was identified, also consisting of just three isolates (Figure 2; Lineage B2). These originated from Zimbabwe (strain F1/06-B21), Sudan (strain F6/05–3) and Uganda (strain Tulya). This latter strain is the *B. abortus* biovar 3 reference (NCTC 10502), though the other two strains in this cluster are described as belonging to biovar 1 in the available metadata. The remaining isolates from Lineage B fell into a larger grouping of 13 strains in total (Figure 2; Lineage B3), which consisted of two lineages (Lineage B3i and B3ii). Lineage B3i was made up of six strains from several locations across SSA, namely Chad (strain 78/14), Nigeria (strain 65/110) and Sudan (strains 87/28, F6/05-2, F6/05-4 and F6/05-9). Clade B3ii consisted of seven strains including a further strain from Chad (strain 80/28) and six from Nigeria (strains babNG1, babNG2, babNG9, babNG15, babNG19, babNG20; described by Suarez-Esquivel et al. (2020)).

The majority of strains included in the wgSNP phylogenetic analysis (390/426; 91.5%) fell within a large clade comprised of two main lineages (Figure 2; Lineage C1 and Lineage C2). Strains included within lineage C1 originated from a broad geographic range across Europe and Asia, covering China (*n* = 9), Georgia (*n* = 7), Greece (*n* = 1), Israel (*n* = 1), Italy (*n* = 8), Netherlands (*n* = 1), Russia (*n* = 23), Spain (*n* = 4) and the United Kingdom (*n* = 3). Clade C1 included biovar reference strains for biovars 5 (strain B3196), 6 (strain 870) and 9 (strain C68; Figure 2; Lineage C1).

The second group within this lineage (Lineage C2) contained the greatest number of strains (331/426; 77.7%) yet was characterised by a relatively low level of diversity, relative to other lineages (Figure 2; Lineage C2). Strains within this clade originated from a broad geographic range across Africa (Egypt, Mozambique and Zimbabwe), Asia (Bangladesh, China, India, Israel, Saudi Arabia and South Korea), Europe (Italy, Kosovo, Poland, Portugal, Republic of Ireland, Russia, Spain and United Kingdom), North America (Costa Rica, United States), Oceania (New Zealand) and South America (Argentina, Bolivia and Columbia). Lineage C2 included the *B. abortus* type strain and biovar 1 reference strain 544^T^ (= NCTC 10093^T^), biovar 2 reference strain 86/8/59 and biovar 4 reference strain 292, as well as *B. abortus* vaccine strains RB51 and S19.

### 3.4. *In silico* multi-locus sequence typing

Multi-locus sequence typing profiles retrieved *in silico* from Ethiopian isolates and other whole genome data (genome assemblies and *de novo* assembled short reads), and profiles retrieved from the PubMLST database, provided a total 650 records for which 9-locus MLST profiles were available, and 628 records for which 21-locus allelic profiles were available. Seven and 38 isolates were not assigned a 9- and 21-locus MLST ST, respectively. Full MLST profiles and metadata are provided in Supplementary Table S5.

#### 3.4.1. 9-locus MLST

**Figure 3:**
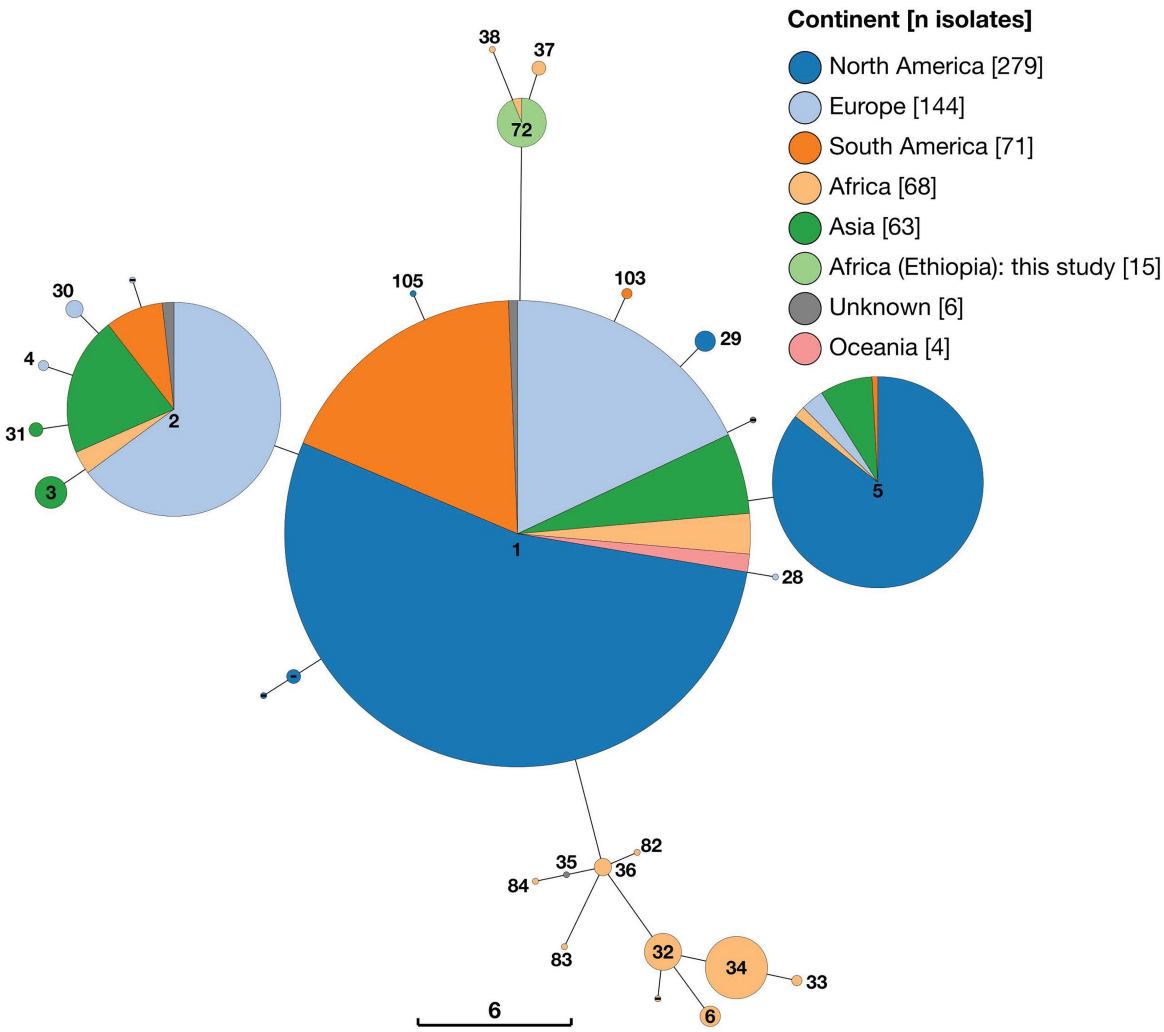
Minimum spaning tree of 650 *Brucella abortus* strains, including 15 Ethiopian isolates, based on 9-locus MLST profiles derived *in silico* from whole genome sequences and downloaded from PubMLST *Brucella* database. Node labels give the assigned sequence type (ST), and node colours give the continent of origin (Ethiopian isolates = ST72). The scale bar gives the number of differing loci.

All Ethiopian isolates were identified as ST72 by 9-locus MLST, an ST which was shared by only a single other strain within the dataset (strain 88/218), isolated from a bovine sample from Mozambique. The two strains identified as belonging to Lineage A by wgSNP (strains 88/217 and 63/294) belonged to 9-locus MLST ST37 and ST38, respectively (Figure 2). Nine-locus MLST profile ST37 was represented by just three strains (88/217, 88/219 and 88/220), all of which arise from the same study in Mozambique, whilst ST38 was represented by only a single strain (63/294), from Kenya. Sequence types ST37, ST38 and ST72 clustered together on a minimum spanning tree based on all 9-locus MLST data (Figure 3). MLST-9 ST72, containing the 15 Ethiopian isolates, branched from ST1 but differed at six of nine alleles.

A second cluster of STs, which also consisted of strains exclusively of sub-Saharan African origin, was evident in minimum spanning tree analysis of 9-locus MLST data (Figure 3). This cluster of STs branched from ST1 but differed by at least four of nine alleles. This cluster consisted of nine separate STs (plus one unassigned ST) from a broad geographic distribution across SSA, including ST6 (Sudan, Uganda and Zimbabwe), ST32 (Cameroon, Chad, Kenya, Nigeria, Rwanda and Tanzania), ST33 (Sudan), ST34 (Chad, Nigeria and Sudan), ST35 (represented by a single isolate of unknown geographic origin [78/36]), ST36 (Nigeria, Senegal, Togo), ST82 (Senegal), ST83 (Niger), ST84 (Senegal). As can be seen in Figure 2, this cluster of STs corresponds to Lineage B in wgSNP analysis.

The majority of strains included in the 9-locus MLST analysis, including all others of sub-Saharan African origin, belonged to three clusters of STs (ST1, ST2 and ST5, and single allele variants of these) which exhibited a broad geographical distribution. The ST1 cluster contained the largest number of strains (*n* = 331), including a large proportion from North America (*n* = 179) and smaller numbers from Europe (*n* = 59), South America (*n* = 60), Asia (*n* = 18), Africa (*n* = 9; Egypt, Mozambique, Zambia, Zimbabwe), Oceania (*n* = 4) and two strains of unknown geographic origin. The ST2 cluster (*n* = 132) was similarly geographically diverse, including strains from Europe (*n* = 80), Asia (*n* = 36), South America (*n* = 10), Africa (*n* = 4; Chad, Egypt, Sudan, Uganda) and two strains of unknown geographic origin. Finally, ST5 (*n* = 112) included strains from North America (*n* = 96), Asia (*n* = 9), Europe (*n* = 4), Africa (*n* = 2; Zimbabwe) and South America (*n* = 1).

#### 3.4.2. 21-locus MLST

**Figure 4:**
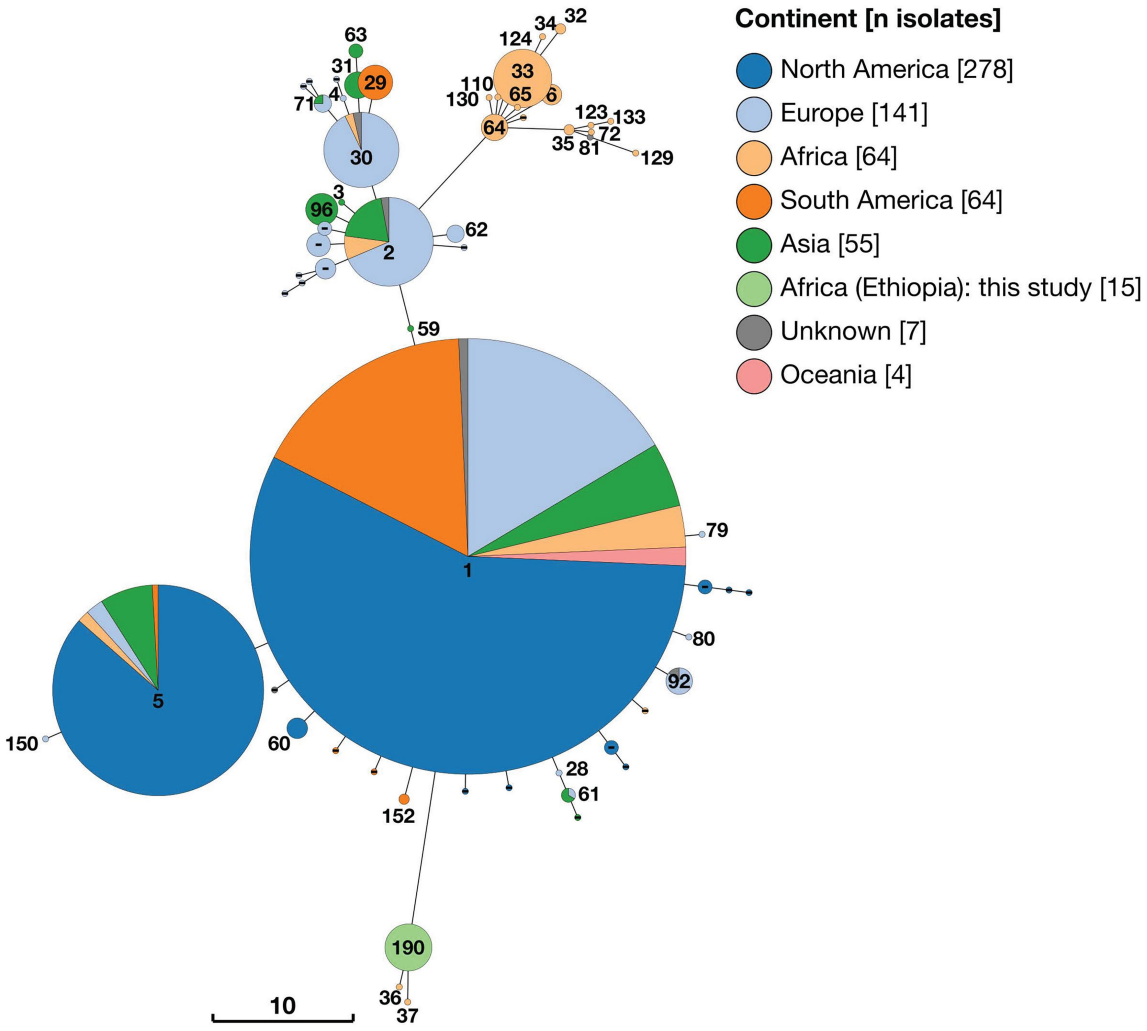
Minimum spaning tree of 628 *Brucella abortus* strains, including 15 Ethiopian isolates, based on 21-locus MLST profiles derived *in silico* from whole genome sequences and downloaded from PubMLST *Brucella* database. Node labels give the assigned sequence type (ST), and node colours give the continent of origin (Ethiopian isolates = ST190). The scale bar gives the number of differing loci.

Analysis of 21-locus MLST data revealed essentially the same pattern as that described above, with the inclusion of additional loci increasing the number of distinct STs identified. Two distinct clusters of STs consisting solely of strains of sub-Saharan African origin were identified, with the majority of strains, including some also from SSA, falling within three larger clusters (Figure 4). The 15 Ethiopian *B. abortus* isolates were assigned to a single ST (ST190), of which they were the only representatives. ST190 clustered with two other STs (ST36 and ST37), each of which was represented by only a single strain (88/217 isolated from Mozambique and 63/294 from Kenya, respectively). These two strains were those identified as belonging to Lineage A by wgSNP (Figure 2). The cluster of 21-locus STs containing the 15 Ethiopian isolates and related strains branched from ST1 but differed at 11 of 21 alleles.

A second cluster of strains (*n* = 46) also originating from SSA was composed of 15 individual sequence types (ST6, ST32, ST33, ST34, ST35, ST64, ST65, ST72, ST81, ST110 ST123, ST124, ST129, ST130 and ST133 plus one isolate with no ST assigned; Figure 4). This cluster of STs corresponded to Lineage B in wgSNP analysis (Figure 2) and originated from 11 countries across SSA (Sudan (*n* = 21), Nigeria (*n* = 8), Senegal (*n* = 4), Chad (*n* = 3), Zimbabwe (*n* = 3), Cameroon (*n* = 1), Kenya (*n* = 1), Niger (*n* = 1), Rwanda (*n* = 1), Togo (*n* = 1) and Uganda (*n* = 1), plus one of unknown origin). This cluster of strains branched from ST2 but differed by at least seven of 21 alleles.

The remaining strains included in 21-locus MLST analysis were assigned to three broad clusters of STs (referred to as ST1, ST2 and ST5 clusters) by minimum spanning tree analysis (Figure 4). In the case of the ST1 cluster, this included 313 strains which were either ST1 (n = 292) or single allele variants of this ST (ST28, ST59, ST60, ST79, ST80, ST92 and ST152), plus a single ST differing by two alleles (ST61). Strains belonging to the ST1 cluster exhibited a broad geographical distribution, originating from North America (*n* = 171), Europe (*n* = 58), South America (*n* = 51), Asia (*n* = 17), Africa (*n* = 9) and Oceania (*n* = 4), plus three from unknown locations. In the case of the ST5 cluster (*n* = 112), the majority of isolates originated from North America (*n* = 96), with smaller numbers of isolates from Asia (*n* = 9), Europe (*n* = 4), Africa (*n* = 2) and South America (*n* = 1). Finally, the ST2 cluster (*n* = 102) consisted of 10 distinct STs, which were either single or double allele variants of ST2 or ST30. These originated from Europe (*n* = 58), Asia (*n* = 28), South America (*n* = 10) and Africa (*n* = 4), plus two isolates from unknown locations.

### 3.5. *In silico* multi-locus VNTR analysis

*In silico* MLVA typing of *B. abortus* from Ethiopia identified heterogeneity amongst the isolates in three of 16 MLVA loci; Bruce04, Bruce 16 and Bruce30 exhibited HGDI estimates of 0.50, 0.73 and 0.50, respectively. These markers are highly-variable micro-satellite loci located in Panel 2B of the MLVA16 scheme (Le Fleche et al., 2006). All other loci were homogeneous within the Ethiopian isolates. Full MLVA profiles and metadata are provided in Supplementary Table S6.

**Figure 5:**
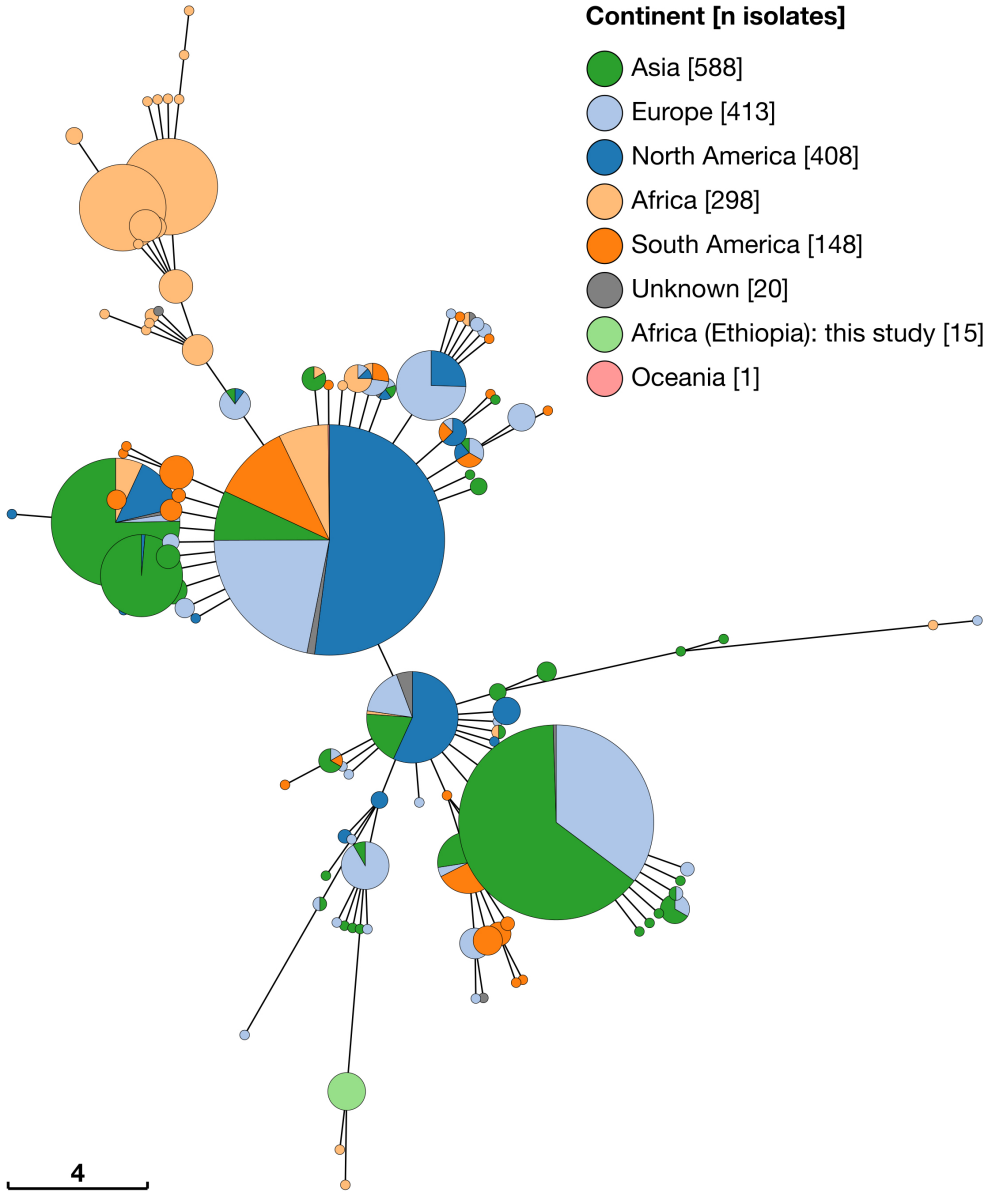
Minimum spaning tree of 1,891 *Brucella abortus* strains, including 15 Ethiopian isolates, based on MLVA11 profiles derived *in silico* from whole genome sequences and downloaded from MLVA-NET *Brucella* database. Node colours give the continent of origin, whilst the scale bar gives the number of differing loci.

Minimum spanning tree analysis was undertaken using a total of 1891 MLVA profiles. In addition to data from 15 Ethiopian isolates this included 147 genome assemblies and 416 short read datasets, downloaded from NCBI and ENA, respectively. Additionally, 1,313 existing *B. abortus* profiles were obtained via the *Brucella* MLVA database. Minimum spanning trees constructed with 11 (excluding Panel 2B) and 16 MLVA loci were broadly consistent in the clustering of isolates (see Figure 5 and Supplementary Figure S1 for MLVA11 and MLVA16 minimum spanning trees, respectively).

MLVA11 minimum spanning tree analysis revealed that the 15 Ethiopian isolates from the current study formed a distinct cluster with two other strains, from Mozambique (strain 88/217) and Kenya (strain 63/294; Figure 5). The Ethiopian cluster was otherwise distinct from its nearest neighbouring cluster by four loci (Bruce08; Bruce11, Bruce42 and Bruce 18). Strains 88/217 and 63/294 were those previously identified as belonging to Lineage A by wgSNP analysis (Figure 2), 9-locus MLST STs 37/38 (Figure 3) and 21-locus MLST STs 36/37 (Figure 4).

The majority of African isolates included within the MLVA11 dataset (230/313, 73.5%) were contained within a second, larger, cluster, comprised of strains originating almost exclusively from SSA. Only a single strain within this cluster was not recorded as originating from the African continent (one isolate of unknown origin [78/36]). African isolates contained within this cluster originated from a broad geographical range across the continent, with significant numbers from West Africa (notably, Senegal [*n* = 139] and Togo [*n* = 28]). The other sub-Saharan African counties represented in this cluster were Cameroon (*n* = 3), Chad (*n* = 4), Guinea (*n* = 2), Guinea-Bissau (*n* = 6), Kenya (*n* = 10), Mauritania (*n* = 1), Niger (*n* = 3), Nigeria (*n* = 8), Rwanda (*n* = 14), Sudan (*n* = 8), Uganda (*n* = 3) and Zimbabwe (*n* = 1). This grouping included the *B. abortus* biovar 3 reference strain (Tulya) which was identified as belonging to wgSNP Lineage B and 9- and 21-locus MLST ST6 (Figures 2–4).

Outside of these two predominantly African groupings, the majority of strains fell within two much larger clusters of related MLVA11 types (Figure 5). The first of these was dominated by a single node of 559 strains sharing the same MLVA11 type, in which North American strains from Costa Rica and the United States formed the majority of isolates (*n* = 291). Within this node 39 strains from the African continent were present, with the majority of these originating from Egypt (*n* = 35) and smaller numbers from Mozambique (*n* = 1) and Zimbabwe (*n* = 3). MLVA11 genotypes associated with this major node (*n* = 429 strains) included 22 strains of African origin, with the majority of these from Egypt (*n* = 19) and a smaller number from Zimbabwe (*n* = 3).

The second major cluster was dominated by a single node of 400 strains, in which strains from Asia comprised the majority of isolates (*n* = 257), but also with a significant number of strains originating from Europe (*n* = 141). MLVA11 genotypes associated with this major node (*n* = 255 strains) included only five strains of African origin.

## 4. Discussion

In the present study we have reported the isolation and first molecular characterisation of *Brucella abortus* from Ethiopia and undertaken a comprehensive analysis of the relationship of these isolates to existing strains. In doing so we have applied a number of molecular typing approaches, in order to maximise the value of existing molecular typing datasets. Despite using different numbers of informative loci, and employing loci with different mechanisms of mutation, the molecular typing approaches applied have generated broadly consistent results. We have shown that the Ethiopian isolates described in the present study expand the known diversity of an early-branching *B. abortus* lineage which previously consisted of only two fully sequenced strains, both of sub-Saharan African origin. A second early branching lineage was also shown to consist solely of strains isolated in sub-Saharan Africa.

Phylogenetic analysis based on wgSNPs identified that Ethiopian *B. abortus* isolates from the current study form a distinct lineage with two previously described strains (88/217 and 63/294 isolated in Mozambique and Kenya, respectively), branching basally to all other isolates included within the analysis. The existence of a basally branching *B. abortus* clade has been previously described, using an expanded 21-locus MLST scheme applied to a comprehensive panel of over 500 *Brucella* isolates (Whatmore et al., 2016). Similarly, Ledwaba et al. (2021) employed wgSNP analysis to describe a similar structure using 175 publicly available *B. abortus* genomes and 13 South African strains. These authors identified major lineages A and B, both of which are described as containing solely African isolates. More recently, Abdel-Glil et al. (2022) presented the results of analyses based on a core genome MLST (cgMLST) scheme for *Brucella*, using 1,764 genes, which also identified three *B. abortus* lineages, with the two most basal lineages represented by strains of African origin.

These results suggest that the ancestral lineage of *B. abortus* may have originated in East Africa, based on currently available information, with the relatively fewer strains of African origin found within the more numerous wgSNP Lineages C1 and C2 potentially reflecting historical re-importation to the continent. A comparable evolutionary scenario has been described for *Mycobacterium bovis*, another zoonotic bacterial pathogen, primarily of cattle. Phylogenetic analyses using whole genome data have indicated that *M. bovis* is likely to have originated in East Africa, and that whilst specific lineages have remained restricted to East and West Africa, others have become globally widely dispersed by cattle movements (Smith et al., 2011; Inlamea et al., 2020; Loiseau et al., 2020). Studies concerning the genetic diversity of *M. bovis* strains present in cattle populations in Ethiopia have demonstrated that two distinct clonal complexes are present, with one of African origin (African 2), and the other reflecting more recent importation of a derived European *M. bovis* lineage (European 3; Almaw et al., 2021).

The present analyses do not allow us to definitively conclude that *B. abortus* Lineage A is ancestral to all other lineages, with an origin in East Africa. It remains possible that more basal lineages exist, and that Lineage A, as currently described, forms part of a larger monophyletic clade. This is particularly true given the relative sparsity of *B. abortus* genome data from Ethiopia, East Africa and the wider continent. It is clear that there is considerable genomic diversity of *B. abortus* within sub-Saharan Africa, and that further sampling across the continent may help to reveal the underlying phylogenetic structure of the species. Future work could also seek to investigate the most likely geographical origin of *B. abortus* lineages using methods to infer ancestral states (e.g., Coscolla et al., 2021). Additionally, phylodynamic analyses, making use of known dates of isolation, may help to infer most recent common ancestors for specific lineages (e.g., Almaw et al., 2021).

The population of Ethiopia has increased markedly in recent decades, with a parallel expansion of the country’s agricultural sector (Firdessa et al., 2012). Ethiopia now has the largest livestock population in Africa, including approximately 65 million cattle, with 70% of the Ethiopian population directly involved in the agricultural sector (CSA, 2020). In the dairy sector this has been associated with a shift from small-scale extensive cattle farming towards larger more intensively farmed herds in urban and peri-urban settings (Firdessa et al., 2012; Deneke et al., 2022). These have been shown to be associated with an increased risk for transmission of infectious diseases such as bovine tuberculosis (Mekonnen et al., 2019). Indeed, previous studies have demonstrated that larger herd size is significantly associated with brucellosis seropositivity in cattle in Ethiopia (Edao et al., 2020). Whilst the present study provides no data regarding prevalence of *B. abortus* in dairy cattle in the Oromia region or in Ethiopia, the isolation of the same strain from multiple animals on a single farm suggests that bovine brucellosis may be a significant problem in similar settings. To date, no brucellosis control strategy, including vaccination, has been implemented in cattle or other livestock species in Ethiopia. As the national herd-level seroprevalence in cattle has been estimated to be 16.3% (Sibhat et al., 2022) it is likely that bovine brucellosis represents an endemic veterinary and public health priority issue in other geographic areas of the country. The successful isolation of *Brucella* from lymph nodes of the mammary glands highlights the potentially significant public health risk from *Brucella abortus* infection in cattle, if milk or dairy products are consumed without pasteurisation. One recent study indicated that the consumption of raw milk was a relatively common practice in Ethiopia, including in the Oromia region, with 20.4% of interviewees consuming raw milk with varying degrees of frequency (Deneke et al., 2022). Furthermore, a much larger proportion of respondents in this study (82%) reporting drinking fermented milk, which is usually made from non-pasteurised or unboiled milk, and which may continue to represent a microbiological hazard despite fermentation (e.g., Zúñiga Estrada et al., 2005).

There are a number of limitations to the present study, which impact the scope of the conclusions which can be drawn. The samples analysed in this work arose from opportunistic sampling of a brucellosis outbreak in a single location in central Ethiopia. As such, it is not possible to draw any conclusions regarding the prevalence of infection within the geographical region (Oromia regional state) or the wider Ethiopian cattle sector. Similarly, the available dataset does not allow us to draw any conclusions regarding the predominant *Brucella* species circulating within the country, and the relative importance of these for livestock and human infections. Appropriately designed prospective epidemiological studies would be required to address these evidence gaps. Furthermore, it is not possible to make any inferences concerning the within-species genetic diversity of other strains of *B. abortus* circulating within Ethiopia. Additional work would be required to characterise diversity within *Brucella* species present in Ethiopia, including sampling of a wider range of livestock hosts and broader geographical coverage across the country.

A methodological issue encountered during the current study was the presence of incorrectly identified genome sequences in open-access genomic databases. A number of recent studies have identified basal clades using *Brucella* sp. genomes which have been incorrectly identified as “*B. abortus*” in public sequence databases. For example, Islam et al., (2019) present a whole genome phylogeny for *B. abortus*, in which strain BCB013 (GCF_000292205.1) is presented as basal to all other *B. abortus* genomes. However, further inspection (e.g., NCBIs Taxonomy Check; Ciufo et al., 2018) reveal that this whole genome sequence is likely to be mis-identified. This is supported by the identification of this assembly, amongst others, for exclusion from the current analysis. Such anomalies highlight the importance of careful curation of datasets downloaded from public repositories, as well as reliable identification of bacteriological isolates, in order to avoid erroneous conclusions from being drawn regarding within-species phylogenetic relationships.

A related methodological issue is the high degree of duplication present in publicly available molecular typing databases. Publicly available databases of both MLST and MLVA data incorporate profiles retrieved *in silico* from genome assembly and short-read datasets (Jolley et al., 2018; Vergnaud et al., 2018). This has resulted in data from individual strains being duplicated, where existing profiles were present from “conventional” (e.g., capillary sequencing based) typing and *in silico* profiles have subsequently been added. This has the potential to artificially inflate the representation of certain strains, potentially skewing the relative composition of specific lineages. Again, this highlights the need for careful curation of datasets downloaded from public repositories.

## 5. Conclusion

The work presented here provides a comprehensive analysis of the phylogenetic structure of *Brucella abortus*, a zoonotic pathogen of global significance. We describe the first detailed characterisation of *B. abortus* isolates from Ethiopia and demonstrate that these isolates expand the known diversity of a basal lineage within the species, which was previously only represented by two isolates from sub-Saharan Africa. By combining analyses based on whole genome sequencing data and existing molecular typing methods (MLST and MLVA) we provide further support for the existence of two basal *B. abortus* lineages, both comprised solely of isolates of sub-Saharan African origin. This work will serve as the basis for further studies concerning the global population structure and evolutionary history of a major zoonotic pathogen which continues to impact the populations of many low- and middle-income countries.

## Data availability statement

The datasets presented in this study can be found in online repositories. The names of the repository/repositories and accession number(s) can be found in the article/Supplementary material.

## Author contributions

BE undertook experimental work, bioinformatic analyses, and contributed to the manuscript. MT supported *post mortem* examinations and sample collection. JW, GA, SB, and AW conceived of and designed the study and contributed to the manuscript. AT performed bioinformatic analyses and contributed to the manuscript. RA performed data analysis and drafted the manuscript. All authors contributed to the article and approved the submitted version.

## Funding

This work was partially funded by the Biotechnology and Biological Sciences Research Council, the Department for International Development, the Economic and Social Research Council, the Medical Research Council, the Natural Environment Research Council and the Defence Science and Technology Laboratory, under the Zoonoses and Emerging Livestock Systems (ZELS) programme (ref.: BB/L018977/1). *Brucellosis* research activities at APHA are supported by the United Kingdom Department for Environment, Food and Rural Affairs (Defra).

## Conflict of interest

The authors declare that the research was conducted in the absence of any commercial or financial relationships that could be construed as a potential conflict of interest.

## Publisher’s note

All claims expressed in this article are solely those of the authors and do not necessarily represent those of their affiliated organizations, or those of the publisher, the editors and the reviewers. Any product that may be evaluated in this article, or claim that may be made by its manufacturer, is not guaranteed or endorsed by the publisher.

## Footnotes

^1^ https://github.com/s-andrews/FastQC

^2^ https://www.ncbi.nlm.nih.gov/assembly

^3^ https://www.ebi.ac.uk/ena/browser/home

^4^ https://nf-co.re/bactmap

^5^ https://github.com/gtonkinhill/pairsnp

^6^ https://github.com/tseemann/mlst

^7^ https://pubmlst.org/brucella/

^8^ https://github.com/dpchris/MLVA

^9^ http://microbesgenotyping.i2bc.paris-saclay.fr
