# Supplementary figures and images for: Whole genome sequencing of Ethiopian Brucella abortus isolates expands the known diversity of an early branching sub-Saharan African lineage
Source: Front Microbiol. 2023 May 4;14:1128966. doi: 10.3389/fmicb.2023.1128966 (PMC10192883; doi:10.3389/fmicb.2023.1128966)

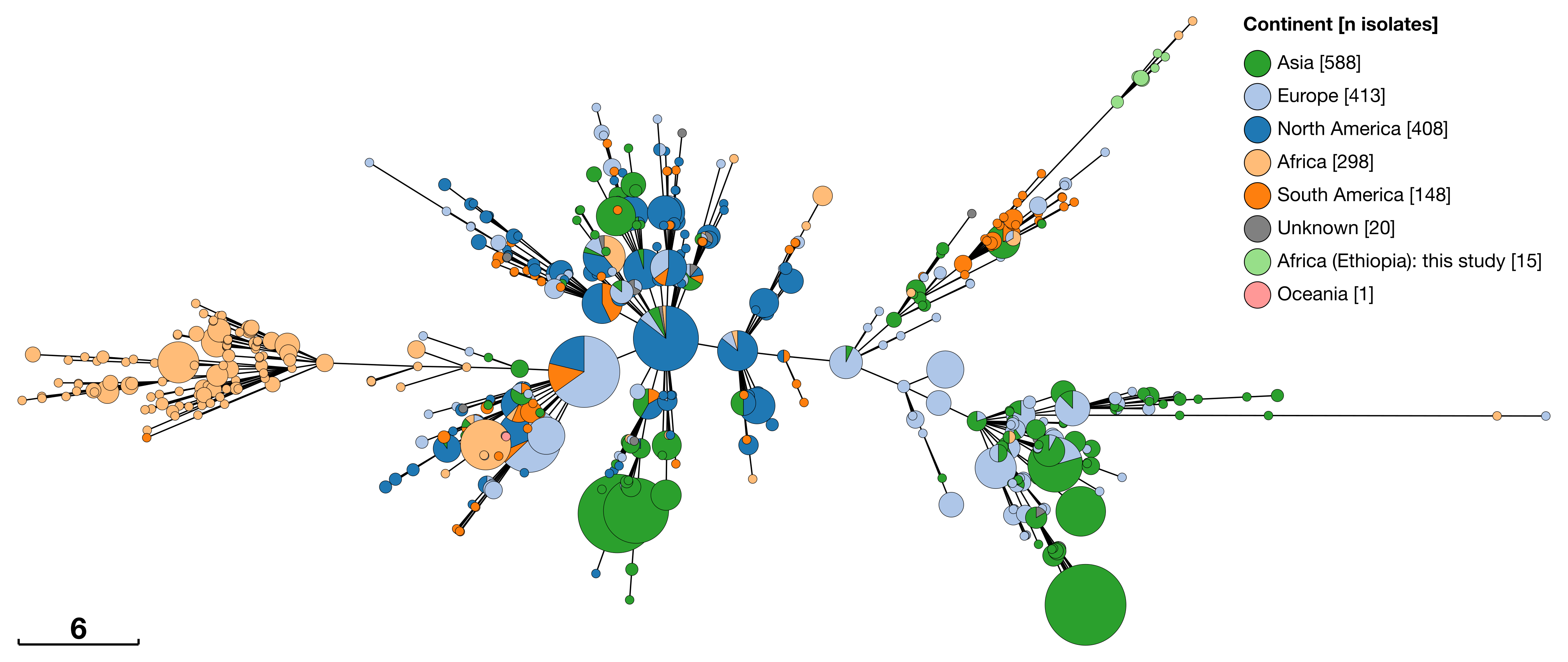

Supplement: Supplementary file 1 [file Image_1.jpg]
