# Supplementary material for: Whole genome sequencing of Ethiopian Brucella abortus isolates expands the known diversity of an early branching sub-Saharan African lineage
Source: Front Microbiol. 2023 May 4;14:1128966. doi: 10.3389/fmicb.2023.1128966 (PMC10192883; doi:10.3389/fmicb.2023.1128966)
Supplement: Supplementary file 3 [file Table_2.docx]

**Supplementary Table S2**

**Table S2.** Whole genome sequencing and d*e novo* assembly statistics for 15 Ethiopian *B. abortus* isolates for which whole genome sequencing data was generated.

|  | **Sequencing** | | **Mapping and alignment** | ***De novo* assembly** | | | |
| --- | --- | --- | --- | --- | --- | --- | --- |
| **Accession number** | **Total reads** | **Reads after QC** | **Average coverage of reference** | **Assembly length (bp)** | **Number of contigs** | **N50** | **GC content (%)** |
| ERS5240204 | 1935708 | 865624 | 65.93 | 3272763 | 37 | 389331 | 57.23 |
| ERS5240205 | 2463478 | 977810 | 74.11 | 3272921 | 38 | 389331 | 57.23 |
| ERS5240206 | 2549826 | 1843206 | 90.12 | 3272978 | 36 | 391359 | 57.23 |
| ERS5240207 | 4134502 | 3066670 | 90.46 | 3272665 | 33 | 389305 | 57.23 |
| ERS5240208 | 1840918 | 1372334 | 90.73 | 3272968 | 38 | 389315 | 57.23 |
| ERS5240209 | 2017458 | 1513806 | 90.62 | 3273657 | 46 | 389323 | 57.23 |
| ERS5240210 | 3652844 | 2693106 | 90.32 | 3272753 | 33 | 391674 | 57.23 |
| ERS5240211 | 3448100 | 1304994 | 92.51 | 3272525 | 32 | 391674 | 57.23 |
| ERS5240212 | 3029680 | 1322114 | 93.56 | 3273635 | 45 | 389315 | 57.23 |
| ERS5240213 | 2792932 | 1199794 | 90.34 | 3273170 | 39 | 389315 | 57.23 |
| ERS5240214 | 3689818 | 1532212 | 93.08 | 3273288 | 37 | 389315 | 57.23 |
| ERS5240215 | 3249690 | 1539940 | 93.99 | 3273651 | 44 | 389323 | 57.23 |
| ERS5240216 | 2454494 | 1060768 | 79.19 | 3272373 | 36 | 389314 | 57.23 |
| ERS5240217 | 2422936 | 1001052 | 75.04 | 3273507 | 43 | 389323 | 57.23 |
| ERS5240218 | 2137504 | 939024 | 70.43 | 3273340 | 39 | 389329 | 57.23 |
